# A Novel Frequency Domain Impedance Sensor with a Perforated Cylinder Coaxial Design for In-Situ Measuring Soil Matric Potential

**DOI:** 10.3390/s19112626

**Published:** 2019-06-10

**Authors:** Chao Chen, Xiaofei Yan, Qiang Xu, Song Yu, Yihan Ma, Xianglin Cheng, Zhongyi Wang, Qiang Cheng

**Affiliations:** 1 College of Information and Electrical Engineering, China Agricultural University, Beijing 100083, China; chern@cau.edu.cn (C.C.); xuqiang@cau.edu.cn (Q.X.); song_y@cau.edu.cn (S.Y.); myh495836915@outlook.com (Y.M.); chengx@cau.edu.cn (X.C.); wzyhl@cau.edu.cn (Z.W.); 2 School of Technology, Beijing Forestry University, Beijing 100083, China; yanxf@bjfu.edu.cn

**Keywords:** soil matric potential, frequency domain, perforated coaxial cylinder, in-situ measurement

## Abstract

Soil matric potential is an important parameter for agricultural and environmental research and applications. In this study, we developed a novel sensor to determine fast and in-situ the soil matric potential. The probe of the soil matric potential sensor comprises a perforated coaxial stainless steel cylinder filled with a porous material (gypsum). With a pre-determined gypsum water retention curve, the probe can determine the gypsum matric potential through measuring its water content. The matric potential of soil surrounding the probe is inferred by the reading of the sensor after the soil reaches a hydraulic equilibrium with the gypsum. The sensor was calibrated by determining the gypsum water retention curve using a pressure plate method and tested in three soil samples with different textures. The results showed that the novel sensor can determine the water retention curves of the three soil samples from saturated to dry when combined with a soil water content sensor. The novel sensor can respond fast to the changes of the soil matric potential due to its small volume. Future research could explore the application for agriculture field crop irrigation.

## 1. Introduction

The soil matric potential is one of the most important parameters in soil science and closely relates to soil water content (*θ*, cm^3^cm^−3^). The soil matric potential is crucial for studying various soil hydrological processes, such as water availability for plants, evapotranspiration, and modeling water and gas flow in partially saturated soil [1]. In addition, it is also applied in many soil studies such as strength and deformation assessment of unsaturated soil and evaluating the physical behavior of different soil water contents [2,3,4].

A pressure plate apparatus is a widespread method used to measure the soil matric potential. It is commonly used to measure the soil water retention curve. However, the pressure plate apparatus is expensive and time-consuming. This method requires several hours or days of equilibration time before performing the measurement of an experimental point. Moreover, it is difficult and inefficient to determinate the soil water matric potential in-situ [5,6].

Dew point potentiometer (Decagon Devices, Inc., Pullman, WA, USA) calculates soil matric potential from the measured dew point and temperature of soil sample [7,8]. This technique is generally used in the laboratory to measure the water potential of disturbed soil samples. It also cannot be used to measure the soil water potential in-situ.

Tensiometer is the instrument commonly used for measuring the soil water potential. It consists of an airtight water-filled tube and a permeable ceramic cup. The ceramic cup can be buried into the soil, and the matric potential of the ceramic equilibrates with that of the soil. This technique is cost-effective. However, tensiometers were unsuited for measurements in dry soils and they were limited in research with the high maintenance [8,9].

The thermal conductivity sensor is an indirect device to measure the soil matric potential. It consists of a heater and porous block and relies on the measurement of the water content of the porous block by measurement of the temperature rise induced by the heater embedded in the block. However, the process of moisture equilibrium between the porous block and the surrounding soil is very slow when the volume of the porous block is quite large [10].

Using the filter paper method to determine the matric potential is also feasible. The soil specimen and filter paper are brought to moisture equilibrium in a constant temperature environment [10]. After the equilibrium is established, the water content of the filter paper is measured. Then, an appropriated filter paper calibration curve obtained from the processes of wetting and drying the filter papers through vapor transfer and the fluid transfer was used to estimate the soil matric potential. However, the equilibration period of calibration or measurement is very long. 

Alternative indirect methods by measuring the electrical resistance within a porous medium that is in hydraulic equilibrium with the surrounding soil are also effective [11]. The Watermark sensor (Irrometer Co., Riverside, CA, USA) was developed for soil matric potential measurement with the internal electrodes measuring the electrical resistance of the reference material [12]. Another relatively new sensor, the MPS-6 sensor (Decagon Devices, Inc., Pullman, WA, USA) consists of a dielectric perforated plate to measure the water content of two ceramic discs with known water retention characteristics [13].

Time-domain matric potential sensors can measure the water content of a porous medium, which is related to matric potential of the sensor through calibration. Or and Wraith (1999) developed and tested a sensor based on time domain reflectometry (TDR) to determine the water content of the porous material, thereby inferring soil matric potential after laboratory calibration [14]. The time-domain matric potential sensors have not been commercialized, and the frequency-domain matric potential sensors are new and have not been widely used.

Rather than using the principle of TDR to measure dielectric permittivity of porous material by measuring the travel time, the objective of this study is to develop a novel sensor which is based on the frequency-domain method for soil matric potential measurement. In order to verify the performance of the sensor, the sensor calibration was conducted under laboratory condition using a pressure plate apparatus. The sensor was tested with three different soil samples to determine the water retention curves.

## 2. Materials and Methods

### 2.1. Probe Design and Construction

**Figure 1:**
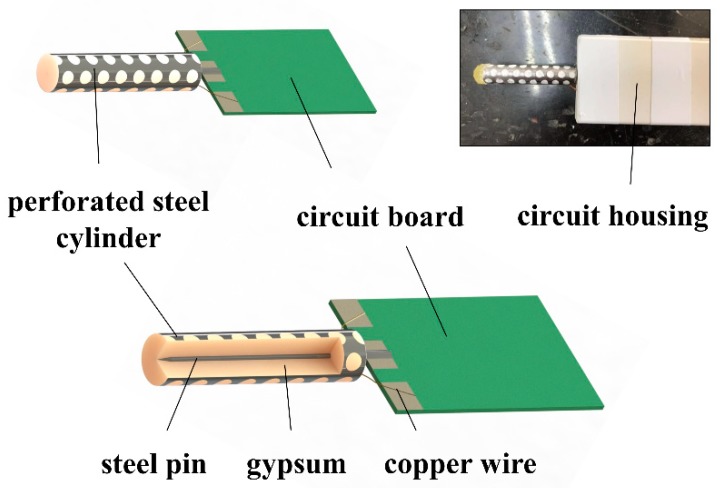
Diagram and photograph of the soil matric potential sensor.

A coaxial probe design for soil matric potential was proposed by Or and Whalley [14,15]. They combine the porous material and stainless steel coaxial cylinder as the probe of soil matric potential sensor. Based on this construction, we developed a more flexible and easier probe to measure the soil matric potential [16]. In this study, a perforated stainless steel coaxial cylinder is used to be the outer electrode, and the inner electrode is a stainless steel pin. The porous material is surrounded by this cylinder, as the steel pin located in the center of the porous material (Figure 1). This probe structure is flexible to change the dimensions and sizes.

The size of the new probe is demonstrated in Figure 1. The length of the whole perforated cylinder is 40 mm, and the diameter is 10 mm. It is smaller than the size of Watermark sensor. The steel pin at the center of the cylinder (diameter: 1 mm, length: 50 mm) is soldered to the emitting end of the circuit board. The stainless steel perforated cylinder (diameter: 10 mm, length: 40 mm) surrounding the porous material is connected to receiving end with copper wires. This steel pin is the emitting electrode, and the perforated cylinder is the receiving electrode. The electrode structure is an innovative design. The porous material is cylindrical gypsum that located inside the steel perforated cylinder. These perforations (diameter: 3 mm) on the wall of the steel cylinder allow water to enter the cylinder and contact with the cylindrical gypsum, then the matric potential of gypsum can be in equilibrium with the surrounding soil. The volume of the porous block in the electrode is about 4 cm^3^, which is smaller than MPS-6 (about 12 cm^3^) [13] and Watermark (about 30 cm^3^) [17] sensors. Therefore, the equilibrium time between the porous material and soil for the proposed sensor is faster than the other two commercial sensors if their porous materials are the same.

### 2.2. Measurement Principle

The matric potential of soil is equal to that of the cylindrical gypsum after hydraulic equilibrium with the surrounding soil. Additionally, the relationship between matric potential and water content of the cylindrical gypsum is previously determined by a pressure plate apparatus. Furthermore, the gypsum water content is converted to the voltage output of the sensor. Therefore, the dielectric measurement of the gypsum can infer the soil matric potential [14].

The principle of measuring gypsum water content is based on a dielectric impedance method that was applied previously [18,19]. The circuit consists of a sinusoidal oscillator, a fixed impedance section, and the perforated cylinder probe which behaves as an additional section of the transmission line with impedance dependent on the dielectric permittivity of the gypsum [10].

The relative permittivity of water (ε_water_/ε_0_ = 81, 20 °C) is much higher than that of the air (ε_air_/ε_0_ = 1) and the gypsum (ε_gypsum_/ε_0_ = 2). The variations of gypsum permittivity surrounding the stainless steel pin cause changes in the impedance (Z_p_, Ω). When this impedance differs from that of the internal impedance (Z_0,_ Ω), a proportion of the signal is reflected back from the junction between the probe and the internal impedance. The difference between the peak voltage at its start and the peak voltage at the junction can be related to the impedance (Z_p_, Ω).

**Figure 2:**
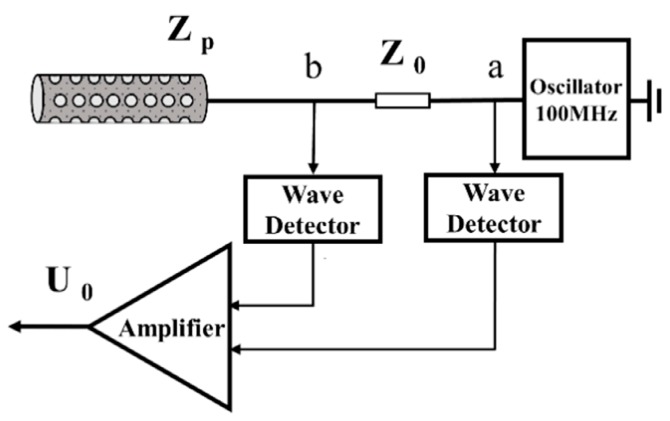
Schematic diagram of measurement circuit, Z_0_, balance impedance, U_a_ and U_b_, output voltage of point a and point b, respectively. U_0_, amplified differential output voltage between point a and point b.

As illustrated in Figure 2, Z_p_ can be expressed by

(1) $$Z_{p}\left(\varepsilon \right)=\frac{Z_{0}}{U_{a}-U_{b}}U_{b}$$

where *ε* is the relative permittivity of wet gypsum, *Z_0_* is a balance impedance(Ω), *U_a_* and *U_b_* represent the output of each wave detector (V). Variations in *Z_p_* can be detected as the voltage difference between two wave detectors (*U_0_*, V) through

(2) $$U_{0}=A_{f}\left(U_{a}-U_{b}\right)$$

(3) $$U_{0}=A_{f}\frac{Z_{0}}{Z_{p}}U_{b}$$

where *A_f_* is the gain of the amplifier. The operating frequency (*f*) in our sensor circuit is set at 100 MHz because the measured water content is relatively independent of porous material electrical conductivity when *f* > 80 MHz [19,20,21]. This circuit requires a stabilized excitation voltage of 5V DC.

### 2.3. Partitioning the Temperature Effect

The output of a dielectric sensor is always significantly affected by temperature [22]. Correction of these effects improves the measurement accuracy of gypsum water content [23]. To eliminate the temperature coefficient of the circuit board, a 10*pf* capacitor insensitive to temperature was connected between the emitter and the receiver as the load of the tested circuit board, replacing the impedance of the gypsum block. This eliminated the temperature-dependency of the permittivity of gypsum water at the probe and allowed correction for only the sensitivity of the circuit board. Then, the circuit board was put in a temperature-controlled incubator, which could be adjusted to different temperatures. The output of the circuit board was recorded as a function of changing the ambient temperature in a temperature-controlled incubator. Further, the temperature coefficient of the circuit board was applied during data processing to correct the relationship between sensor output and temperature [16].

Besides, the permittivity of water in gypsum is also a temperature factor affect the output of the dielectric sensor. To eliminate temperature effects on the permittivity of water in gypsum material, we combined a permittivity model incorporating temperature effects on porous material [24]:

(4) $$\varepsilon^{\alpha }=\theta_{l}\varepsilon_{l}{\left(T\right)}^{\alpha }+\theta_{s}\varepsilon_{s}^{\alpha }+\theta_{a}\varepsilon_{a}^{\alpha }$$

(5) $$1=\theta_{l}+\theta_{s}+\theta_{a}$$

with a similar expression for the permittivity of free water [25]:

(6) $$\varepsilon_{l}\left(T\right)=78.54\left[\begin{array}{c} 1-4.579\times 10^{-3}\left(T-25\right)+1.19\times 10^{-5}{\left(T-25\right)}^{2}-\\ 2.80\times 10^{-8}{\left(T-25\right)}^{3} \end{array}\right]$$

where *ε* is the permittivity, *θ* is of the volumetric of the fraction of each component in the porous material, and the associated subscripts *l*, *s* and *a* refer to water, solid component, and air, respectively. Additionally, *α* is an empirical calibration exponent and can be determined [26].

### 2.4. Sensor Calibration

To obtain the relationship between the output of the novel sensor and soil matric potential, a calibration was carried out using a pressure plate in the laboratory. The novel sensor was buried completely in the wetted sandy loam soil.

Establishing the relationship between the sensor output and the matric potential requires the apparatus to set a certain value. The pressure plate apparatus was adapted to accommodate the sensor so that the relationship of the sensor output and the applied pressure could be obtained [27,28]. Sensor output (U_1_/mv) was measured at each applied pressure (P, kPa) until equilibrium was reached. After reaching equilibrium between air pressure and matric potential of the gypsum inside the probe, each sensor reading at a respective equilibrium pressure gave a certain point of the calibration curve. Equilibrium status was reached when there was no water outflow from the apparatus. Then, we recorded and calculated the average of three sensors’ voltage outputs.

The outputs and pressure plate readings were monitored over three-month period time at room temperature (20 ± 5^o^C). The output of the completely saturated sensor was obtained after immersing the soil water matric potential probe in water. Then, a power function of the form

(7) $$\psi \left(U_{1}\right)/kPa=a\cdot {\left(U_{1}/mV\right)}^{b}$$

was used as a calibration function. It is a simplified equation from the Campbell model of the soil water retention curves [29]. This function was chosen as an example of a traditional approach, where measured data pairs serve as a basis for fitting a calibration function and mentioned by Nolz et al. [28].

### 2.5. Testing of the Novel Sensor in Laboratory

**Figure 3:**
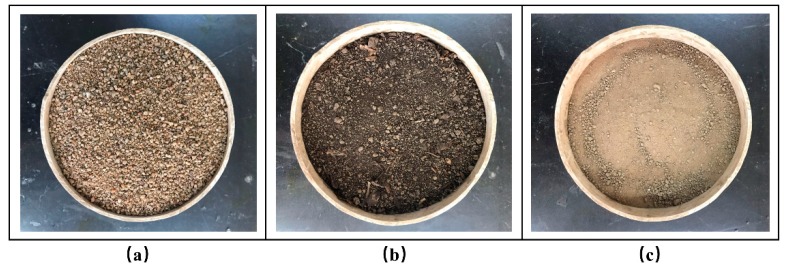
Soil samples of different textures: (**a**) Sandy soil; (**b**) forest humus; (**c**) sandy loam.

Three materials of different textures, sandy soil (sand: 90%, silt: 3.5%, clay: 6.5%) and forest humus and sandy loam (sand: 42.1%, silt: 44.2%, clay: 13.7%) were used for this test, as shown in Figure 3. The three soil samples were sieved with a 2-mm mesh screen. The sandy soil and sandy loam were oven-dried at 105 °C for two days. The samples of forest humus were oven-dried at 65 °C for two days [30]. Three types of soil were sieved and dried, target and reference soil were scaled in three identical containers (diameter: 20 cm, height: 9 cm) with different bulk density measured as the relationship between the weight and volume of the soil samples. The initial dry bulk density was 1.25 g cm^−3^, 0.57 g cm^−3^, 1.13 g cm^−3^ for the sandy soil, the forest humus and the sandy loam, respectively. The simplified evaporation from wet to low moisture ranges was used as a standard laboratory method to determinate the soil water matric potential. These soil samples were slowly saturated with distilled water in the laboratory (20 ± 2 °C). Once the soil was saturated, the novel sensor was buried into these soil samples to monitor the changes of soil water matric potential during drying.

**Figure 4:**
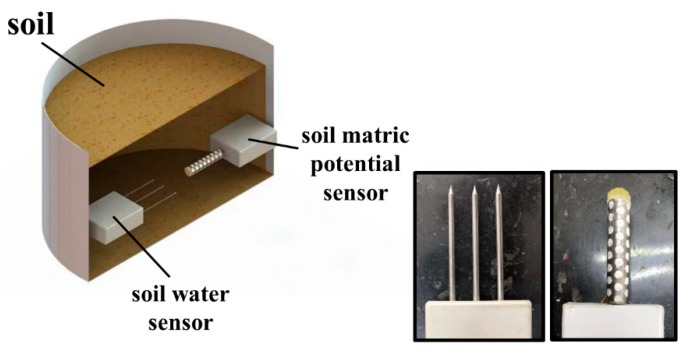
Diagram of the experiment.

Furthermore, as shown in Figure 4, a three-pin FDS100 soil water content sensor (Unism, Inc., Beijing, China) is used to determine the change of soil moisture. Then, the relationships between soil water content and soil water matric potential of three materials of different texture can be obtained. These relationship curves are termed the soil water retention curves, which combine the water content and the matric potential of soil. These curves are important soil functions with several agriculture applications. They can apply in many soil studies such as strength and deformation assessment of unsaturated soil and evaluating the physical behavior of different soil water content. Further, the retention curves of the soils were modeled using the multimodal approach proposed by van Genuchten: [31].

(8) $$\theta =\theta_{r}+\frac{\left(\theta_{s}-\theta_{r}\right)}{{\left[1+{\left(\alpha h\right)}^{n}\right]}^{m}}$$

where $m=1-\frac{1}{n}$, *α* and *n* are empirical fitting coefficients, *h* is matric potential, and *θ _s_* and *θ _r_* are the saturation and residual water contents, respectively.

## 3. Results and Discussion

### 3.1. Temperature Correction

**Figure 5:**
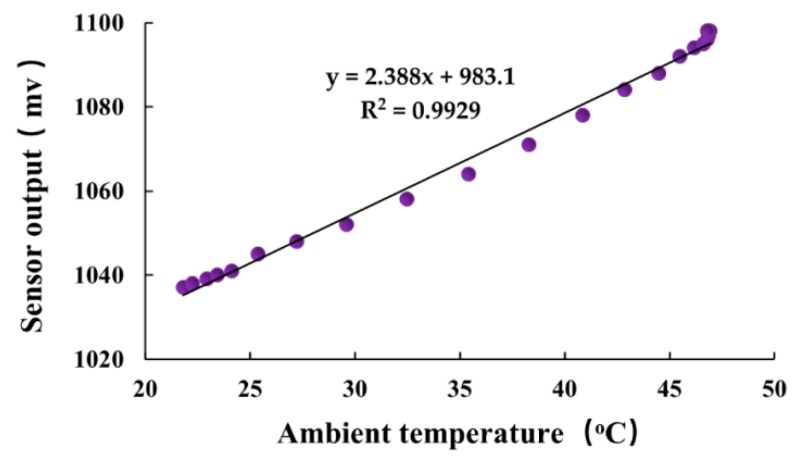
Relationship between the sensor output and ambient temperature.

Figure 5 shows the relationship between the outputs of the circuit board and ambient temperature. Over an operating range of 20–45 °C, the positive temperature coefficient was well-described by a linear regression equation with high R^2^ (0.9929). The linear regression suggested a useful temperature coefficient to partially correct the measurement using the dielectric sensor. This relationship allowed precise temperature correction of sensor outputs. The corrected outputs were acquired by first correcting the temperature coefficient of the circuit board using the equation yielded in Figure 5, and then correcting temperature-dependent permittivity of water in gypsum with Equation (4) and Equation (5). After temperature correction, the accuracy of the sensor measurement can be improved. The temperature correcting procedure in detail can be seen in the literature [16].

### 3.2. The Results of Calibration

**Figure 6:**
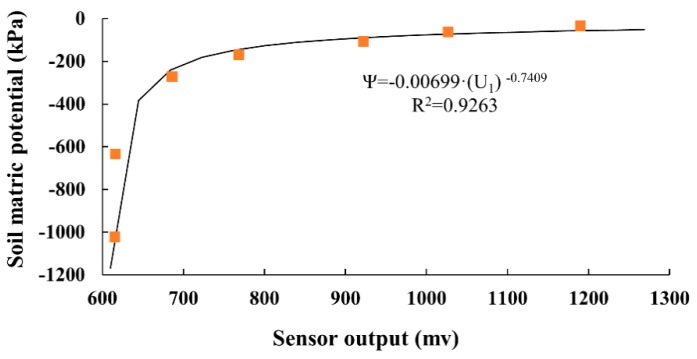
Curve between sensor output and soil matric potential.

The novel sensor was used in the pressure plate apparatus and it took several days for us to be certain that the output had reached an equilibrium value at each pressure. The results of calibration establish the relationship between sensor output and pressure value. When the equilibrium pressure is 0 kPa, the gypsum medium is saturated and the novel sensor reading is 1270 mV. The sensor reading is nearly 1200 mV when the equilibrium pressure ranges from 0 to 20 kPa. As the equilibrium pressure increases, the sensor readings within the pressure plate apparatus decrease. The water in the gypsum is continuously discharged as the equilibrium pressure increases. When the pressure plate apparatus reading is nearly 200 kPa, the sensor output is 701 mV. The lowest reading of this sensor is 615 mV when the pressure value becomes 1000 kPa, and the water content of the gypsum does not change anymore. Then, the main drying curve of the gypsum can be determined. Figure 6 shows the curve of the sensor output (U_1_) and the matric potential transformed by the pressure. 

The Campbell model and the van Genuchten model used power function to fit the soil water retention curve, and thus, a power function of the form $\psi =a\cdot U_{1}{}^{b}$ with the coefficients a = −0.00699 and b = −0.7409 turned out to give the best correlation (coefficient of determination R^2^ = 0.9263).

### 3.3. Results of the Testing in Laboratory

**Figure 7:**
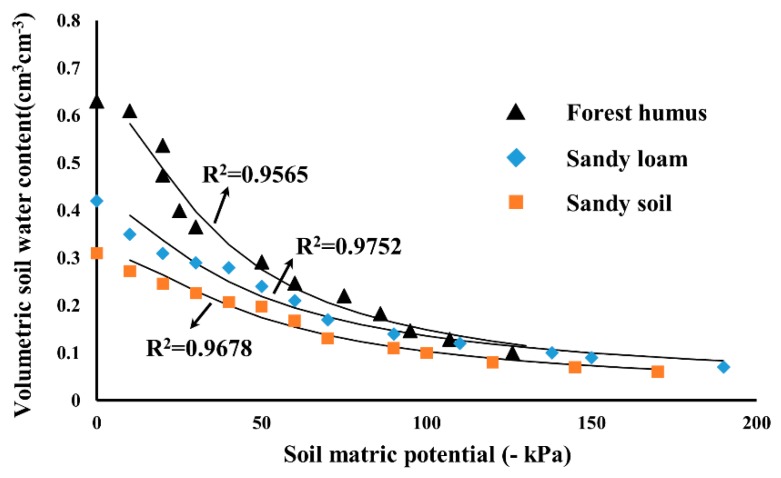
Water retention curves measured with the novel sensor on three different soils.

The soil water retention curves measured with two sensors for sandy soil and sandy loam and forest humus are shown in Figure 7. The soil water matric potential is obtained from the readings of the novel sensor. The soil water content is inferring from the three-pin soil water content sensor. The parameters of the water retention curves estimated with the van Genuchten model are summarized in Table 1, as well as the determination coefficient (R^2^) for the comparison between the measured and modeled water retention curves for the three soils.

From these curves, it can be generally seen the forest humus has the highest saturated moisture of about 0.63 cm^3^cm^−3^. The saturated volumetric water content of sandy soil was nearly 0.3 cm^3^cm^−3^, and the sandy loam was nearly 0.4 cm^3^cm^−3^. This is due to the higher organic matter in forest humus. With the range of −100~0 kPa, the forest humus has more water storage under the same water potential. In comparison to the sandy soil and sandy loam, the water-retention characteristic of sandy loam has good performance. These differences should be attributed to the different textural characteristics. Additionally, the pores of sandy soil are larger than sand-loam, the water in these pores will be drained first during the drying process. When their soil water content approached the lowest, the soil matric potential values of three different soils decreased significantly.

## 4. Conclusions

In this paper, we proposed a novel sensor to measure the soil water matric potential based on the FD method. The soil water matric potential was determined by the matric probe using the gypsum as the porous material. The utility of the sensor was demonstrated by investigating three soil samples of different texture in laboratory. The suitability of the novel sensor for describing changes in soil water retention characteristics associated with alterations in soil structure is of great interest in soil structure studies. More research is needed to explore the ideal porous materials with a wide range of water retention and the hysteresis effects on the relationship of the porous materials. Future research will be extended to field experiments.

## Figures and Tables

**Table 1:** The measured saturated (*θ_s_*) and residual (*θ_r_*) volumetric water content, and the empirical fitting coefficients *α*, *n* for the water retention curves corresponding to van Genuchten model, and the correlation coefficient (R^2^) for the comparison between the measured and modeled water retention curves.

Parameters	Sandy Soil	Sandy Loam	Forest Humus
θ_s_	0.31	0.42	0.63
θ_r_	0.06	0.07	0.09
n	1.93	1.80	2.00
α	0.0306	0.0387	0.0409
R^2^	0.97	0.97	0.96
